# Supplementary material for: Potential implications of rising sea level on American Horseshoe Crab (Limulus polyphemus) spawning beaches in two Florida counties
Source: PLoS One. 2025 Nov 5;20(11):e0333812. doi: 10.1371/journal.pone.0333812 (PMC12588468; doi:10.1371/journal.pone.0333812)
Supplement: S1 Fig — (PDF) [file pone.0333812.s001.pdf]

Horseshoe Crab Survey

Map location*

The location of the horseshoe crab sighting. Enter location by either:

1. Clicking the "Find my location" icon

on the left hand side of the map viewer.

2. Zooming in and dragging the map so that the bottom point of the blue marker is over the location where the sighting occurred. **Note: the marker stays in the center while the map moves underneath.**

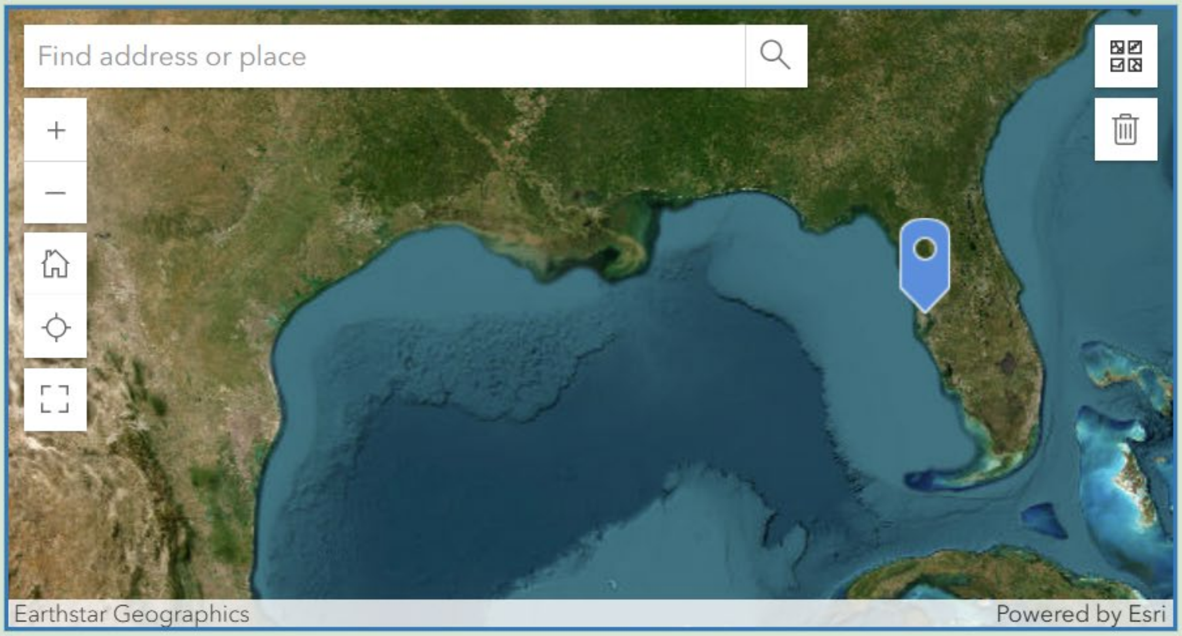

Lat: 27.906843 ° Lon: -82.664307 °

Location description*

(city, beach name; please indicate any other details that might be important)

County of sighting*

-Please select-

Date of sighting*

MM/DD/YYYY

Time of sighting*

-Please select-

Number of Horseshoe Crabs*

Estimates are okay

Sex

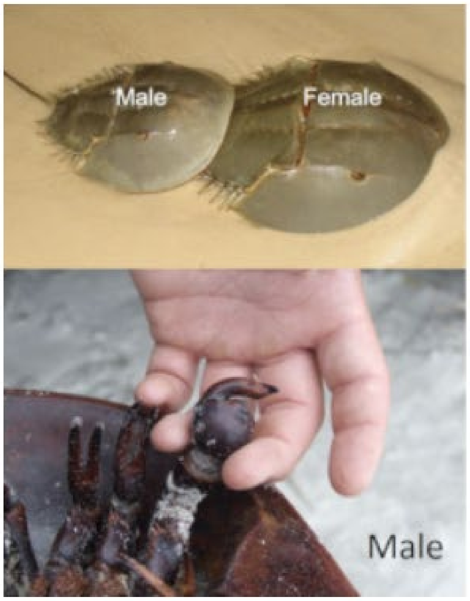

Number of females

123

Number of males

123

Were the crabs mating?

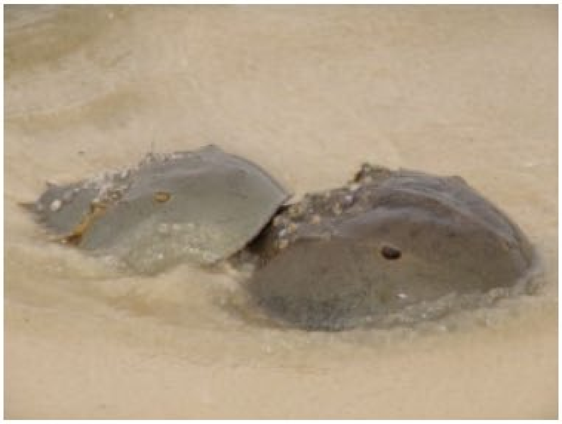

(i.e. two or more crabs attached)

-Please select-

Did you observe any juvenile crabs?

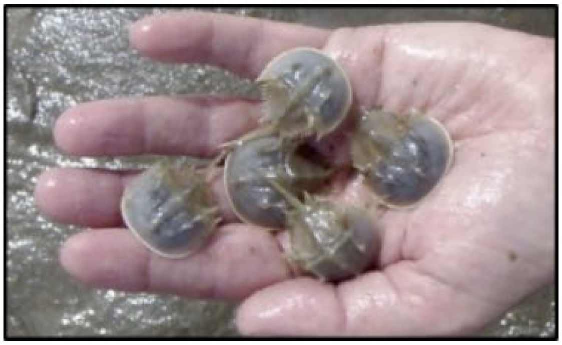

(about 4 inches wide or smaller)

-Please select-

Did you observe any dead crabs?

-Please select-

Was it windy or calm out when you observed the horeshoe crabs?

-Please select-

Additional comments

Please note characteristics of the habitat, tides, horseshoe crab behavior, etc.

May we contact you if we have questions about your sighting?*

-Please select-

Please make sure you have entered coordinates for your sighting.

Submit
