# Supplementary material for: Potential implications of rising sea level on American Horseshoe Crab (Limulus polyphemus) spawning beaches in two Florida counties
Source: PLoS One. 2025 Nov 5;20(11):e0333812. doi: 10.1371/journal.pone.0333812 (PMC12588468; doi:10.1371/journal.pone.0333812)
Supplement: Attachment — Submitted filename: PONE-D-24-52275_Review_comments.docx [file pone.0333812.s004.docx]

PONE-D-24-52275

Potential Implications of Rising Sea Level on Nesting Horseshoe Crab Populations in Two Florida Counties

PLOS ONE

Dear Dr. Heres,

Thank you for submitting your manuscript to PLOS ONE. After careful consideration, we feel that it has merit but does not fully meet PLOS ONE’s publication criteria as it currently stands. Therefore, we invite you to submit a revised version of the manuscript that addresses the points raised during the review process.

==============================

Dear Authors,

The reviewers have provided their comments and suggestions for the manuscript. Please carefully address each of their points, revise the manuscript accordingly, and submit your responses to their comments.

Be sure to:

- Indicate which changes you require for acceptance versus which changes you recommend
- Address any conflicts between the reviews so that it's clear which advice the authors should follow
- Provide specific feedback from your evaluation of the manuscript

==============================

Please submit your revised manuscript by Jan 27 2025 11:59PM. If you will need more time than this to complete your revisions, please reply to this message or contact the journal office at [plosone@plos.org](mailto:plosone@plos.org). When you're ready to submit your revision, log on to <https://www.editorialmanager.com/pone/> and select the 'Submissions Needing Revision' folder to locate your manuscript file.

Please include the following items when submitting your revised manuscript:

- A rebuttal letter that responds to each point raised by the academic editor and reviewer(s). You should upload this letter as a separate file labeled 'Response to Reviewers'.
- A marked-up copy of your manuscript that highlights changes made to the original version. You should upload this as a separate file labeled 'Revised Manuscript with Track Changes'.
- An unmarked version of your revised paper without tracked changes. You should upload this as a separate file labeled 'Manuscript'.

If you would like to make changes to your financial disclosure, please include your updated statement in your cover letter. Guidelines for resubmitting your figure files are available below the reviewer comments at the end of this letter.

If applicable, we recommend that you deposit your laboratory protocols in [protocols.io](http://protocols.io/) to enhance the reproducibility of your results. Protocols.io assigns your protocol its own identifier (DOI) so that it can be cited independently in the future. For instructions see: [https://journals.plos.org/plosone/s/submission-guidelines#loc-laboratory-protocols](https://track.editorialmanager.com/CL0/https:%2F%2Fjournals.plos.org%2Fplosone%2Fs%2Fsubmission-guidelines%23loc-laboratory-protocols/1/010f0193c02721c5-454d12e4-b5b9-4e27-ba44-2237fe959925-000000/1nqGuqJ7UfzsUo1ERsRmeA-GhirsnBhb7ZVWsT8cVVM=189). Additionally, PLOS ONE offers an option for publishing peer-reviewed Lab Protocol articles, which describe protocols hosted on [protocols.io](http://protocols.io/). Read more information on sharing protocols at [https://plos.org/protocols?utm_medium=editorial-email&utm_source=authorletters&utm_campaign=protocols](https://track.editorialmanager.com/CL0/https:%2F%2Fplos.org%2Fprotocols%3Futm_medium=editorial-email%26utm_source=authorletters%26utm_campaign=protocols/1/010f0193c02721c5-454d12e4-b5b9-4e27-ba44-2237fe959925-000000/BzuGmfg1NYYpdw9EvDmnsBx1uKv6hXcfLDIOIeuPpcw=189).

We look forward to receiving your revised manuscript.

Kind regards,

Phuping Sucharitakul
Academic Editor
PLOS ONE

Journal requirements:

When submitting your revision, we need you to address these additional requirements.

1. Please ensure that your manuscript meets PLOS ONE's style requirements, including those for file naming. The PLOS ONE style templates can be found at

<https://journals.plos.org/plosone/s/file?id=wjVg/PLOSOne_formatting_sample_main_body.pdf> and

<https://journals.plos.org/plosone/s/file?id=ba62/PLOSOne_formatting_sample_title_authors_affiliations.pdf>

2. Please note that PLOS ONE has specific guidelines on code sharing for submissions in which author-generated code underpins the findings in the manuscript. In these cases, we expect all author-generated code to be made available without restrictions upon publication of the work. Please review our guidelines at <https://journals.plos.org/plosone/s/materials-and-software-sharing#loc-sharing-code> and ensure that your code is shared in a way that follows best practice and facilitates reproducibility and reuse.

3. We note that the grant information you provided in the ‘Funding Information’ and ‘Financial Disclosure’ sections do not match.

When you resubmit, please ensure that you provide the correct grant numbers for the awards you received for your study in the ‘Funding Information’ section.

4. We note that Figures 1, 2 and 4 in your submission contain [map/satellite] images which may be copyrighted. All PLOS content is published under the Creative Commons Attribution License (CC BY 4.0), which means that the manuscript, images, and Supporting Information files will be freely available online, and any third party is permitted to access, download, copy, distribute, and use these materials in any way, even commercially, with proper attribution. For these reasons, we cannot publish previously copyrighted maps or satellite images created using proprietary data, such as Google software (Google Maps, Street View, and Earth). For more information, see our copyright guidelines: <http://journals.plos.org/plosone/s/licenses-and-copyright>.

We require you to either (1) present written permission from the copyright holder to publish these figures specifically under the CC BY 4.0 license, or (2) remove the figures from your submission:

1. You may seek permission from the original copyright holder of Figures 1, 2 and 4 to publish the content specifically under the CC BY 4.0 license.

We recommend that you contact the original copyright holder with the Content Permission Form (<http://journals.plos.org/plosone/s/file?id=7c09/content-permission-form.pdf>) and the following text:

“I request permission for the open-access journal PLOS ONE to publish XXX under the Creative Commons Attribution License (CCAL) CC BY 4.0 (<http://creativecommons.org/licenses/by/4.0/>). Please be aware that this license allows unrestricted use and distribution, even commercially, by third parties. Please reply and provide explicit written permission to publish XXX under a CC BY license and complete the attached form.”

Please upload the completed Content Permission Form or other proof of granted permissions as an "Other" file with your submission.

In the figure caption of the copyrighted figure, please include the following text: “Reprinted from [ref] under a CC BY license, with permission from [name of publisher], original copyright [original copyright year].”

2. If you are unable to obtain permission from the original copyright holder to publish these figures under the CC BY 4.0 license or if the copyright holder’s requirements are incompatible with the CC BY 4.0 license, please either i) remove the figure or ii) supply a replacement figure that complies with the CC BY 4.0 license. Please check copyright information on all replacement figures and update the figure caption with source information. If applicable, please specify in the figure caption text when a figure is similar but not identical to the original image and is therefore for illustrative purposes only.

The following resources for replacing copyrighted map figures may be helpful:

USGS National Map Viewer (public domain): <http://viewer.nationalmap.gov/viewer/>

The Gateway to Astronaut Photography of Earth (public domain): <http://eol.jsc.nasa.gov/sseop/clickmap/>

Maps at the CIA (public domain): <https://www.cia.gov/library/publications/the-world-factbook/index.html> and <https://www.cia.gov/library/publications/cia-maps-publications/index.html>

NASA Earth Observatory (public domain): <http://earthobservatory.nasa.gov/>

Landsat: <http://landsat.visibleearth.nasa.gov/>

USGS EROS (Earth Resources Observatory and Science (EROS) Center) (public domain): [http://eros.usgs.gov/#](http://eros.usgs.gov/)

Natural Earth (public domain): <http://www.naturalearthdata.com/>

5. We note that there is identifying data in the Supporting Information file < HSC data from Survey123 website (2009 - 2023) copy.xlsx>. Due to the inclusion of these potentially identifying data, we have removed this file from your file inventory. Prior to sharing human research participant data, authors should consult with an ethics committee to ensure data are shared in accordance with participant consent and all applicable local laws.

Data sharing should never compromise participant privacy. It is therefore not appropriate to publicly share personally identifiable data on human research participants. The following are examples of data that should not be shared:

-Name, initials, physical address

-Ages more specific than whole numbers

-Internet protocol (IP) address

-Specific dates (birth dates, death dates, examination dates, etc.)

-Contact information such as phone number or email address

-Location data

-ID numbers that seem specific (long numbers, include initials, titled “Hospital ID”) rather than random (small numbers in numerical order)

Data that are not directly identifying may also be inappropriate to share, as in combination they can become identifying. For example, data collected from a small group of participants, vulnerable populations, or private groups should not be shared if they involve indirect identifiers (such as sex, ethnicity, location, etc.) that may risk the identification of study participants.

Additional guidance on preparing raw data for publication can be found in our Data Policy (<https://journals.plos.org/plosone/s/data-availability#loc-human-research-participant-data-and-other-sensitive-data>) and in the following article: <http://www.bmj.com/content/340/bmj.c181.long>.

Please remove or anonymize all personal information (<specific identifying information in file to be removed>), ensure that the data shared are in accordance with participant consent, and re-upload a fully anonymized data set. Please note that spreadsheet columns with personal information must be removed and not hidden as all hidden columns will appear in the published file.

[Note: HTML markup is below. Please do not edit.]

Reviewers' comments:

Reviewer's Responses to Questions

**Comments to the Author**

1. Is the manuscript technically sound, and do the data support the conclusions?

The manuscript must describe a technically sound piece of scientific research with data that supports the conclusions. Experiments must have been conducted rigorously, with appropriate controls, replication, and sample sizes. The conclusions must be drawn appropriately based on the data presented.

Reviewer #1: No

Reviewer #2: Partly

2. Has the statistical analysis been performed appropriately and rigorously?

Reviewer #1: I Don't Know

Reviewer #2: No

3. Have the authors made all data underlying the findings in their manuscript fully available?

The [PLOS Data policy](http://track.editorialmanager.com/CL0/http:%2F%2Fwww.plosone.org%2Fstatic%2Fpolicies.action%23sharing/1/010f0193c02721c5-454d12e4-b5b9-4e27-ba44-2237fe959925-000000/0uG9IwC6zGT4OatjTnwmjMWWhjhaVIn0DKo2askr6To=189) requires authors to make all data underlying the findings described in their manuscript fully available without restriction, with rare exception (please refer to the Data Availability Statement in the manuscript PDF file). The data should be provided as part of the manuscript or its supporting information, or deposited to a public repository. For example, in addition to summary statistics, the data points behind means, medians and variance measures should be available. If there are restrictions on publicly sharing data—e.g. participant privacy or use of data from a third party—those must be specified.

Reviewer #1: Yes

Reviewer #2: Yes

4. Is the manuscript presented in an intelligible fashion and written in standard English?

PLOS ONE does not copyedit accepted manuscripts, so the language in submitted articles must be clear, correct, and unambiguous. Any typographical or grammatical errors should be corrected at revision, so please note any specific errors here.

Reviewer #1: No

Reviewer #2: No

5. Review Comments to the Author

Please use the space provided to explain your answers to the questions above. You may also include additional comments for the author, including concerns about dual publication, research ethics, or publication ethics. (Please upload your review as an attachment if it exceeds 20,000 characters)

Reviewer #1: This manuscript by Contrada et al. seeks to assess “the potential impact of sea-level rise on horseshoe crab nesting habitats”. The authors do this by combining sightings of horseshoe crab spawning with GIS characterization of how shorelines differ across sea level rise scenarios, based on current habitat characterizations. The findings may be relevant if presented in the proper contexts and if the authors restrict their discussion to the results of their analyses. The methods of the paper are also unclear, but I think they can be improved with greater organization and a clearer workflow of the process. My specific comments below are meant to be helpful in providing constructive feedback to improve this manuscript.We appreciate your thoughtful review and comments, we think that we have addressed them thoroughly and feel the manuscript has improved.

• L27: There are no data on living shorelines presented in this paper to support this conclusion. Please remove. References to living shorelines were removed, except for mentions in the discussion as ways to preserve sandy shorelines.
• L40: Add “hereafter ‘horseshoe crab’” so the reader knows that you will be using that name to refer to this species.This has been modified, and “American” was removed in subsequent mentions.
• L51: Which changes are the authors referring to when they mention “these changes”? Please be specific about which changes you are referencing. Specific changes were added to the text.
• L90: Only the rufa subspecies relies on horseshoe crab eggs for their migration.Scientific name updated to reflect the *rufa* subspecies.

• L103: Please clarify what the symbol “±” is meant to represent. Presumably this is a measure of error, but is it the standard error of the mean, or some other measure?

It represents standard deviation

• L105: Please provide a link or another citation so readers know how to locate this information. The information was used based on a previous reviewer recommendation, this reference is no longer needed.
• L120-123: The font in this section is different than surrounding text. Font size has been corrected
• L142-143: This sentence indicates that the present study used only data from 2017-2023, but the sentence before includes reference to a figure with data from 2002-2023. Please clarify the use of these two datasets in the present study (i.e., how and why the data from 2002-2023 are used, and how and why the data from 2017-2023 are used. This was included in teh methods to justify and explain the use of 2017-2023 data. “Beginning in 2017, observations of horseshoe crabs reported through online surveys and Survey 123 which have included GPS locations; therefore, in the present study, observations collected through these methods from 2017–2023 were chosen based on their location accuracy”

• L143-144: Please provide specifics on the type and timing of imagery used to develop to ESI layers used in your analysis. This is important context for understanding when your habitat characterizations took place as compared to when your horseshoe crab sighting information was collected.

Year and citation for published maps are included, and we have added context here “ESI Shorelines are classified by map developers through a combination of overflights, aerial photography, remotely sensed data, ground truthing, and existing maps and data spanning the previous 10 years”

• 143-155: Maybe create a separate section for habitat designation (e.g., ‘Habitat Characterization’, or something similar) where you can describe the ESI? Right now this information is included in the section of ‘horseshoe crab nesting data’.

Added at line 159-169

• L170-180: This paragraph would also fit better in a ‘Habitat Characterization’ section.

Moved
• L191-194: Please include specific information on which analyses were used.

If the reviewer is referring to the analyses used to predict sea level rise, we recommend the citation as the source of that information as it is complex and outside of the scope of this paper to attempt to fully explain in summary.
• L189-196: This section currently comes after the sentence stating that “habitats ranked “zero”, which classified areas where nesting was possible, were used for further analysis”. So were the horseshoe crab reports discussed here (L189-196) all of the horseshoe crab reports, or just the ones ranked as zero? I think this is referring to all the horseshoe crab reports, but the way this is currently written I am unsure. Please clarify your workflow. To clarify this, we moved the section describing ranking of horseshoe crab sighting reports and TAble 1, to the “Horseshoe Crab spawning data” subsection.

• L230: Kernal density analysis is not discussed in your methods. Please include information in your methods and workflow regarding what this analysis is and how it was conducted.

We agree this deserves clarification and added a comprehensive citation on the methods.
• L248: Kernal density is again mentioned here but not in the Methods, please address.

We agree this deserves clarification and added a comprehensive citation on the methods

• L238-241, L255-259, L280-282: Why are figure captions located in the middle of the text? This is formatted to PlosOne requirements.
• L308: Please clarify why is meant by ‘economic success of horseshoe crabs’. This text was adjusting to clarify and changed to “ecological and economic contributions”
• L310: Changing ‘shift’ to ‘loss’ would be more accurate. The results quantified the potential loss of habitats. A ‘shift’ in habitat would suggest that you quantified how habitat changed from one habitat to another.

While we understand the reviewers viewpoint, we can not definitively say that the habitat would be lost since horseshoe crabs use multiple types of habitat so it may or may not be a loss which is why we chose “shift”.
• L348-360: The results provide no information on horseshoe crab eggs or their use by predators such as shorebirds. This section of the discussion is not relevant, but

We removed this section.

L337-339: Redundant with introduction. This section was modified to reduce redundancy with introduction.

• L373: The authors here acknowledge that they did not “examine potential habitat shifts”, an important point that I am glad they acknowledge here. I implore the authors to review their own language throughout the rest of the manuscript to be clear that they are not suggesting this elsewhere in the manuscript.

We are not, we understand the risk of confusion but believe that it is disingenuous to assume that habitat would be strictly lost and so shift is the better term.

• L405-427: Living shorelines are not in the results of the manuscript. They are not tested experimentally nor do they appear to be listed as one of the habitat types in the analyses. The discussion of living shorelines thus serves purely a review of published studies, none of which appear to be specifically related to horseshoe crab habitat. As such, this section is not relevant to the results of the study and should be removed from the manuscript. We drastically modified this section to move away from discussion of something outside of the scope of this study. A few sentences remain around L402.

• L429: You use the term ‘horseshoe crab’ throughout the manuscript to refer to L. polyphemus, but in this instance you are using ‘horseshoe crab’ to refer to a broader taxonomic group of horseshoe crabs over millions of years. This should be clarified. Maybe you could change this to say ‘Xiphosurid horseshoe crabs have adapted to changing environmental conditions over millions of years’? The intention of ‘horseshoe crab’ in this context was still *L. polyphemus,* we felt that adding reference to Xiphosurid would confuse readers, as it has not been previously introduced.
• L434-435: This statement is inconsistent with the limitations described by the authors. Please re-frame this statement to acknowledge the uncertainties around how these findings might be used by managers. We feel that the statement “practical approach to identifying areas with high concentrations of species that rely on shoreline habitats for nesting. Managers can use these projections to predict critical nesting areas that are likely to shift over the next 100 years. “ is within the scope of the paper.

• L441: Is there acknowledgement of the funds that supported personnel to conduct this work or to collect horseshoe crab data? Funding information has been added.
• L452: Publication is missing References have been checked and anything missing was added.
• L518: Is this a publication or a website? More information is needed. Website reference was added.
• L615-617: This is not peer-reviewed. Please cite peer-reviewed study. Maybe this one?
<https://esajournals.onlinelibrary.wiley.com/doi/full/10.1002/fee.2738> Thank you, the peer reviewed manuscript was added.
• Figures are blurry and hard to read. Figures will be high resolution with publication, waiting on ESRI copyright.
• Figure 5: Please add a x-axis with values We experimented with adding an x-axis with values but felt that it was visually unappealing and busy . Because we are presenting the bars with the proportions labeled, we felt it sufficient given that proportions indicate that the sum must be 100.
• Figures 6 & 7. These are difficult to view for color-blind readers These figures have been changed to better facilitate ADA compliance.

Reviewer #2: Synopsis
In this manuscript, the authors characterized the possible change in nesting habitat at where American horseshoe crab were observed nesting in FL. Using crowd-sourced, community science data, the authors generated a dataset of HSC observations, assigned landcover classifications where HSC were observed, and quantified loss of landcover classifications at three future timesteps. The authors found habitat where HSC were observed in 2017 and 2020 was predicted to be lost due to anticipated sea level rise. The authors propose that living shoreLmay be a more suitable climate adaptation than hardened structures, such as sea walls, as living shoreLare also beneficial for spawning HSC. I see value to this work and highly encourage the authors to pursue revision of this work.
In the manuscript’s current form, there is inconsistent and unclear use of terminology that makes methodology unclear, and additional clarification is necessary to fully comprehend the study findings. Below, I’ve attempted to provide thorough feedback in the spirit of facilitating revision.Thank you for your thoughtful and constructive review of the manuscript. We aimed to make as many changes as possible and believe this is now a better manuscript.

General feedback
-Consider writing in first person (e.g., we studied, we collected data, we analyzed, we found) throughout.

A fair suggestion but the first author preferred to keep as is.

-Consistency in terminology: Throughout the manuscript, there’s inconsistent use of terminology that makes it difficult to follow the authors’ message. Some terms, like landward and seaward, should be clarified on their first use. There’s inconsistent formatting of name of horseshoe crab-consider dropping American after first use. Please consider using anthropogenic instead of ‘man-made’ throughout. There’s discussion on habitat shift, though what a ‘shift’ is was not defined, and there’s contradictory statements in the manuscript as to whether evaluating shifts occurred (see L337-374 vs L116-118, 126-129, 170-172, 308-311; I do not believe ‘shifts’ were measured, as this, to me, suggests an area of landcover classification x transforming to landcover classification y, though the methods are unclear). Please consider using change instead of loss when quantifying the area of landcover classifications predicted to be affected by sea level rise.

We include the term shift as well as loss, because both are taking place. We know definitively that as the sea level rises what habitat was used will be lost, that habitat is now inundated, we are not ruling out that new habitat (shift) may become available, only that we know what is here now will be gone. We have gotten mixed opinions from internal and external peer-reviews, and feel that we can only make about 50% happy. We hope to have increased consistency of terminology throughout the manuscript, even if we are not using ‘change’.

-Introductory content: I found it unusual to read a horseshoe crab paper that does not use the word ‘spawning’ to describe horseshoe crab breeding behavior. I recommend using the word ‘spawning,’ when referring to active breeding behaviors, and ‘nests,’ to refer to the locations where HSC eggs are deposited. These changes were made throughout the manuscript.

-Methods and habitat selection/preference: This manuscript presents a characterization of the areas where horseshoe crabs were reported. There is no use vs availability analysis, which is an analysis frequently used to explore organismal habitat selection (and preference). Without a use vs availability analysis or a similar statistical analysis, the use of ‘habitat selection’ or ‘preference’ is inappropriate. Line 361 was the reference to potential habitat preference, this was adjusted to reflect the lack of use vs. availability analyses.

. -Community science: There’s a lot of excellent value from crowd-sourcing data collection, though there can be some caveats to consider. As written, I’m unclear on the potential caveats and more information is needed to understand the data that was submitted by community members. I’ve outlined some points for consideration in the manuscript. The use of citizen-science in the manuscript may not have been the most appropriate word choice, as the data was from public reports. We have a citizen-science program that surveys regularly at locations identified from the public reports, thus, we used them interchangeably. These spawning reports are specific to public reported data. We have clarified that in the methods and discussion.
--Consider adding a paragraph later in the Introduction that explains the HSC community science program in FL, then get into the details of this program in the Methods. It would be helpful to describe the value of using community science data in evaluating species distributions. The end of the Introduction would likewise be an excellent place to mention the value of the community-derived HSC database. Based on the previous comment and the modifications that were made, this should no longer be a point of confusion.

--What is meant by ‘horseshoe crab activity’ (L134-135) This is an error, we intended it to say ‘spawning’? Does this mean that when community members saw a spawning horseshoe crab (as suggested by header on line 133), they reported, or was the data portal intended for any observation of a crab? If observations were of any crab, how was nesting distinguished from a dying or deceased crab on the beach, or a crab that may be embedded in the substrate between tides?the reporting is specific to spawning crabs, but there is also the opportunity to record dead or juvenile crabs if they are present.
--Were observations only of marked crabs (e.g., <https://www.fws.gov/project/horseshoe-crab-cooperative-tagging-program>) or did observations also include unmarked crabs? If the dataset included unmarked crabs, how did the authors control for the potential of repeat observations? Observations were not associated with tagged/marked crabs, only specific to the presence of spawning horseshoe crabs. We adjusted and clarified the Spawning Data in the methods which clarifies this question--Were there efforts to control for sampling effort (frequency of reporting) which could vary based on popularity of the beach with recreationists/community members, or in areas that may be more popular with nature-minded individuals that would be more inclined to use a wildlife reporting app? The frequency of reports were not part of the analyses, more to classify on presence/absence of spawning and the associated habitat. strictly presence/absence data, density is relative to the number of sightings that occur in one place not the number of crabs observed. These data are separate from the Florida horseshoe crab watch program which follows a more rigorous method of data collection. The data in this study are opportunistically collected data submitted by the untrained public, but we provide imagery to better guide the public on what is and isn’t a spawning horseshoe crab. We concede that these data are not of high quality compared to data collected by trained scientists but would rank it similar to that of the ebird dataset which has been used in peer reviewed publications hundreds of times, using similar methods. We also added to the discussion “This may indicate a potential habitat preference difference between the populations or reflec the habitat associated with public access, and thus more often reported”, as an explanation that data will be somewhat reflective of public access or desire to report.

--What training resources were available for the public? For example, the data include fields for HSC sex, which may not be readily apparent to community members. Please consider explaining signage, visuals of the app as an appendix, and other resources that community members could use when submitting a report.We will add this as an appendix or link to survey, as the journal allows. Within the survey are pictures and descriptions to help the public accurately identify the sex, spawning, etc. <https://survey123.arcgis.com/share/e7946f74bcda43128eea82690b95047a?sm=f8QN2JV2JBVp4TIiJ61IE7WsMBGDooQ2rzmR8u47ugM%3d?sm=f8QN2JV2JBVp4TIiJ61IE7WsMBGDooQ2rzmR8u47ugM%3d>

Will include screenshots of website as supplemental material.

-Use of citations: Some sentences in the manuscript appear to be missing citations (e.g., L69-70). Citation added. Numerous review articles are cited in lieu of the primary sources; please consider citing primary articles. Some citations appear to be used incorrectly (e.g., reference [21] in L92-94). Please consider a careful review of references to ensure all are used appropriately. Per your suggestion, citations were reviewed and additions and modifications were made.

Specific feedback
-L1-2: The use of populations in the title suggests an evaluation of demographic impacts, which were not part of the study objectives. Not evaluating demography is not a detractor, though perhaps a recast to reflect that this manuscript evaluates projected habitat changes for spawning HSC would be more appropriate. Consider including full study organism name (American horseshoe crab) and Latin binomial epithet. Title adjusted to “Potential Implications of Rising Sea Level on American Horseshoe Crab Spawning Beaches in Two Florida Counties”.
-L12: Sentence starts with a value-laden word. Consider replacing ‘unfortunately’ with ‘Though.’ This has been changed to “Though”
-L13 and throughout: Lowercase H and C in horseshoe crab. After first mentions of American horseshoe crab in Abstract and Introduction, consider consistently using ‘horseshoe crab.’ This has been changed throughout the manuscript.
-L17 and throughout: Consider replacing ‘habitat type’ from ESI products with ‘landcover classification,’ re: Krausman, P. R. and M. L. Morrison. 2016. Another plea for standard terminology. The Journal of Wildlife Management 80(7):1143-1144. doi: 10.1002/jwmg.21121
We agree with the reviewer’s concern for consistent terminology and have made some changes in the manuscript to reflect that but we feel that habitat is more apt terminology since we are speaking directly about verified horseshoe crab spawning locations.

-L32-41: This paragraph about climate change indicates that sea level rise will affect coastal ecosystems, but does not provide detail on what changes sea level rise is predicted to cause. Describing anticipated consequences from sea level rise will be important for understanding the potential implications for HSC.

We do not know all of the predicted effects of sea level rise and the literature is ever changing and depends heavily on human influence. We made the decision to focus only on the evidence that there would be a change, not what that change entails.
-L36-38: Consider removing ‘in response to the threat of rising sea levels,’ as the starting clause of the sentence already indicates this. Thank you, this has been deleted.
-L42 vs 148: rip rap vs riprap. Changed to riprap
-L49-50: HSC also breed along the Yucatan Peninsula of Mexico, though the Mexican population is genetically distinct (García-Enríquez, J. M. et al. 2023. Genetic study of the American horseshoe crab throughout its Mexican distribution. Conservation and management implications. Biodiversity and Conservation 32:489-507. doi: 10.1007/s10531-022-02508-4).Thank you for including this citation, it has been added to the manuscript.
-L58-59: The background information on HSC breeding ecology is scattered in the Introduction. It would be helpful to describe HSC life history, spawning behavior, and development in a single, cohesive paragraph, and this location would be an appropriate location.

More details about horseshoe crab life history added in this location.
-L59-61: Consider a recast for this sentence. Many organisms will breed/reproduce in suboptimal areas if preferred habitat is unavailable (Fretwell, S. D. 1972. Populations in a seasonal environment. Princeton University Press.) and there’s a lot of work in understanding population density, age, and other factors in habitat preference and habitat selection. Perhaps recast to focus on habitat features that facilitate HSC spawning and embryonic development. Consider moving a paragraph from the Discussion (habitat shifts paragraph on L382-404) into the Intro, which feels more appropriate when considering beneficial habitat features for HSC

We agree that there is much more to learn about what will happen regarding shifts in habitat, our primary goal with this manuscript was to definitively say there will be a shift, but what shift we can not say, and how horseshoe crabs adapt we can only allude to what may happen. We describe features that facilitate spawning in the introduction very intensively and we discuss the potential shift in the discussion because it is the “after” or result of the study. Before we did our analysis we did not know there would be a shift so it isn’t appropriate to pull it into the introduction.
-L65-67: Consider adding “respectively” after 0.3 mm [12] to clarify measures correspond to each state. Respectively has been added.
-L90: Consider Red Knot Calidris canutus rufa, the subspecies that migrates from South America to Arctic Canada and relies on HSC eggs in DE bay. Thank you, the species name has been updated.

-L92-94: There’s limited evidence to suggest HSC are a major dietary item of sea turtles since the 1980s. The reference used here [21] does not support HSC are a dietary item of sea turtles. Ehlinger and Tankersley ( Ehlinger GS, Tankersley RA. 2007. Reproductive ecology of the American Horseshoe Crab Limulus polyphemus in the Indian River Lagoon: An overview. Florida Scientist 2007;70(4): 449–463. ) does suggest this, but is based on the citation (botton and Ropes 1987), which does not reference sea turtle consumption. Therefore, we will remove this reference. We were surprised by this revelation.

-L96-112: While indicating HSC importance is valuable, this paragraph contains a lot of information on the economic value of HSC, which feels tangential to the study goals. Consider shortening this section to succinctly highlight the economic value of HSC, and incorporate threats to HSC.

We agree it was excessive so we removed some of the information in this section.
-L99: Consider Latin binomial epithets for American eel and whelk.The scientific names have been added
-L103: Please clarify measure after average-is this SD, SE? This information was removed in response to another reviewer, no longer needs changing

-L105: Citation should be numeric. ASMFC written out and numeric citation added.
-L109: Remove space between ‘sea-’ and ‘level’ corrected
-L115: Study does not truly evaluate habitats, as this suggests a statistical comparison. Instead, the manuscript presents a characterization of the habitats and simulates a projected change at three distinct times. Please consider recasting to clarify this is a descriptive study.

Changed language to reflect this.
-L116-118: Why were these years chosen? Please consider including a rationale for these years.

Added rationale to methods section
-L119: Introduction seems to end abruptly. Please consider including statements on the importance of this work for FL, and how these results could extend elsewhere along the Gulf and Atlantic coasts.

Text added for broader impact.

-Methods line 120: Consider placing study site description before statement on ArcGIS-i.e., reorganize Methods with L125-132, L133-168, then include L121-123 between L169-170.

This change was made along with a subsection title change suggested by another reviewer.

-L121: Add manufacturer information (e.g., Esri, Redlands, CA, USA). manufacturer added

-L126: Remove Latin epithet for horseshoe crabs. removed

-L130: Replace text citation with numeric. Citation was placed there in error. Fixed

-L126-132: Please consider adding additional detail on study sites, as this will be useful for others in both contextualizing findings and understanding how results here may extend elsewhere. Consider adding approximate coordinates for locations (can be centered in county) for readers. Consider describing the usage of shorelines-how much of the area is protected (e.g., wildlife refuges), what’s the ratio of protected to unprotected areas (e.g., space center in Brevard county). Additionally, this study includes areas on the Gulf and Atlantic coasts, which may have very different temperatures, currents, and habitats. The authors may consider framing this study as an Atlantic coast/Gulf coast comparison, which may expand the interest of readers outside FL.

We like the idea of this, but would consider it better suited to a completely different study. Our study was limited to where we had the largest number of horseshoe crab reports, and we do not consider horseshoe crabs found in the Indian River Lagoon to be substantially different in behavior from gulf coast horseshoe crabs, if anything they are more similar to gulf coast crabs in behavior than those in the north atlantic. We would be happy to discuss this more with the reviewer because it is a very interesting line of thought but not suited to the theme of this manuscript.
--Relatedly in this section, please consider a separate map that shows study areas, and limit locations on the map to only include names. The information displayed in Figure 1 is a result and would be more suitable in the Results section.

Figure 1 is not meant as a result, it is reflective of the data already collected through public reports, rather than explicitly for this study.
--Consider explaining how sea level rise is expected to affect the barrier islands in the study areas. Are barrier islands migrating, changing?

While a very interesting line of thought, it is outside the scope of our study, we are focused on shorelines mapped by the ESI index.

-L146-147: A brief explanation of how coastal habitat designations were made in ESI would be useful. E.g., were these designations the result of supervised classifications in ArcGIS, were they Descriptions of habitat designations and citations were added.

-L134-135, Figure 1: Shows a result, would be more appropriate to reference in the Results section and not in Methods.

We consider this data to be preliminary, as it is associated with the FWC public reporting data that are generated regularly for reporting purposes.
-L134-135: Who were results reported to (i.e. which agency)? How were people able to complete the form (e.g., was there signage, or did people have to know of the reporting system) and how did they know how to fill out fields in the form? Understanding the resources and training available to respondents is necessary for understanding the validity of behavioral observations that include fields for HSC sex. Are these of marked HSC (e.g., <https://www.fws.gov/project/horseshoe-crab-cooperative-tagging-program>)? Are reports of HSC standardized to control for ‘sampling effort,’-e.g., I would a priori predict more reports of HSC in areas with more people. If HSC are unmarked, how might the possibility of repeated observations of the same individual in areas with high human traffic skew interpretation of results?

All excellent questions, we are using this as strictly presence/absence data, density is relative to the number of sightings that occur in one place not the number of crabs observed. These data are separate from the Florida horseshoe crab watch program which follows a more rigorous method of data collection. The data in this study are opportunistically collected data submitted by the untrained public, but we provide imagery to better guide the public on what is and isn’t a spawning horseshoe crab. We concede that these data are not of high quality compared to data collected by trained scientists but would rank it similar to that of the ebird dataset which has been used in peer reviewed publications hundreds of times, using similar methods.

-L138: How was it determined if an observation was ‘robust’?Wording here was changed to better reflect that these data were more location specific.
-L141: Include a reference for kernel density analysis. Added

-L142: How was subtidal nesting ruled out from the data? From a quick look at the data, I did not see a field explicitly for spawning/nesting. How was it determined if an observation was below the water line? This is especially important, as tideLcan change rapidly within a year, and vary drastically between years. Suboptimal vs. optimal spawning beach. We needed bounds to the analyses and made the decision that best reflects studies conducted on horseshoe crab spawning spawning in Florida, which is surrounding the Mean HIgh Water Line (MHWL)
-L143-149: Additional information describing the ESI dataset is necessary. How did NOAA designate landcover classifications? Please consider including a table that lists the landcover classifications (habitat types) and a description of that classification.

Added additional details to describe ESI dataset.

-L160-161: Were in-person or spatial assessments of HSC observations at high tide or low tide? Were assessments made at the same year as the reported observation? How did the authors account for potential changing shorelines? We provided imagery in the survey of paired horseshoe crab and horseshoe crab are not found on the shoreline out of the water if they are not mating so we can safely assume they were actively spawning if seen on the shoreline paired up.
-L165-166: It’s unclear what kinds of landcover classifications were considered, and so it remains unclear what habitats were considered likely, unlikely, or unknown nesting substrates. Please include a table of landcover classifications, a description of that classification, and sources that support assignments in the ranking system.

This would add a significant number of tables, figures, and text, which is why we included the ESI reference that goes into substantial detail of creation and classifications.

-L178-180: It was surprising to see HSC do not nest on FL’s Atlantic and Gulf coast facing beaches; elsewhere on the North Atlantic, HSC nesting can occur on Atlantic beaches. A citation to help justify the exclusion of these beaches in this study would be useful.

We are simply using the data gathered by the public for our analysis and horseshoe crabs are not seen spawning outside of the Indian river lagoon often or at all on the Atlantic beaches. This is an area worthy of more research but none exists as far as we are aware. We suspect it has to do with the wave action, but again more research is warranted.

-L182, 206: There are two subheaders for ‘Sea-Level Rise Scenarios,’ in the Methods section.

Second instance changed to Digital Elevation Data.
-L186-188: The Atlantic Coast and Gulf Coast are anticipated to experience differing extents of sea level rise. Were the study areas evaluated under the same scenario, or were scenarios spatially explicit? They were evaluated under the same scenario of 0.5 m sea-level rise (line 233), the use of the Digital elevation model accounts for the slope of beaches and thus accounts for different extents of sea level rise based on geographic location.

-L190-193: The explanation behind the choice to use 0.6 m wide polygons suggests a possible misinterpretation of sea level rise. If I understand, text appears to suggest that a 0.6 m strip is a conservative boundary that encompasses shoreline habitats that will be affected by a 0.5 m sea level rise. However, this would imply that a 0.5 m sea level rise will result in a waterline shift of 0.5 horizontal m across the beach, when in practicality, the waterline should shift 0.5 m in elevation. To understand the land area affected by the elevational change of the waterline, analyzing the DEM is necessary to determine that line.

The 0.6 m buffer is a representation of the area surrounding the MHWL, representing optimal spawning sites for horseshoe crabs. This does not describe the area that would be impacted most by sea level rise, rather an area by which to calculate a loss or shift. We agree that the waterline could shift on a low sloping sandy beach, but in Florida and the habitats described here, may be directly adjacent to habitat horseshoe crabs are unable to spawn. The DEM is used in conjunction with the scenarios described in the manuscript.

-L199-204: I’m unclear what additional information is gained from the inclusion of nesting habitat slope. Was there one DEM available to quantify slope, and if so, how did the authors consider the potential for annual variation in beach slope and can slope be reasonably extrapolated to 2040, 2070, and 2100 to inform estimates of habitat change due to sea level rise? Were slope estimates averaged across all habitat types in an area? It’s also impossible to glean HSC selection of slope in this study without a use vs availability analysis. Slope was used to compare these habitats to existing literature on horseshoe crab nesting preference. We did not use it as a parameter to determine the slope after sea level rise scenarios were implemented.

-L207-209: Consider moving reference to the digital elevation model to line 199, which is the first reference of the DEM in the Methods. Greater description of the DEM would be useful.

We included more citations and details.

-L210-212: It’s unclear if the timesteps reflect a 2D area of change (‘subtracting’ the area anticipated to be lost to sea level rise from the coast at time step t) vs a 3D area of change that considers the volume of water and the beach slope. Additional clarification is necessary to understand how pixels were considered ‘underwater’ at each of the three timesteps.

Raster data is three dimensional so it is not simply adding .5 meters to the current high tide line, but considering the shape of the area being inundated and correcting accordingly.
-L214: Remove extra apostrophe between quotes of “Erase” fixed
-L217-218: Consider omitting how percent is calculated.Was requested by previous reviews so keeping, but we agree and have removed.
-~L222: Consider providing some general summary statistics about the study areas-lengths of shorelines, annual areas of landcover classifications. Consider also including summary statistics on community science reporting, which could be used to explain Figure 1 (vs in the Methods). On average, how many observations are made per year (± SD)? In Fig 1, how many years of data are shown? Would results change if data were presented as mean annual crab sightings? The Introduction notes two distinct spawning periods for FL HSC (Feb-May, Aug-Nov; L84-85), which is very unique. Please consider describing temporal variability in HSC nests observations.

We clarify in the manuscript that the community science data (wrong terminology on our part) was not used, rather public reporting data, a similar but different program. This clarification should satisfy this comment and need for descriptive statistics.
-L225, 243: Separating results into counties makes this paper very FL-specific, and difficult to glean generalities that extend beyond FL. Consider recasting to focus on Gulf coast vs Atlantic coast, and characterize the habitats available on each coast.

. Florida coastline is very diverse and the analyses of two counties may not appropriately represent coastwide scenarios. Given this, we kept it county specific, which also reflects the best data available, but would love to expand the research to other areas. Similar data doesn’t exist for other states at this time.
-L233-236, 250-253: How were these habitats (landcover classifications) delineated? This wasn’t clear in the Methods It is described in the ESI citations. It would add several figures and paragraphs of text, so we felt it best to cite.
-Fig 2, 4: I note that some of these ‘clusters’ overlap with densely populated or popular areas (e.g., St Petersburg). How were data handled to account for sampling effort? Consider combining this figure into one with two panels. We did not account for sampling effort. We address the deficiencies of the data in the limitations section.
-Fig 3, 5: These show percents, not proportions. Consider combining this figure into one with two panels. The graphs and associated text were modified to reflect proportions.
-Table 2, 3: Consider including a starting area of the landcover classification from 2017/2020. As an example, the projected loss of 94.8% of mangroves appears alarming, though may be of minimal ecological consequence if the starting area of mangroves represented an insignificant area in 2020. It would not make graphic sense to do so as it would look like Figure 1.
-Results: Consider including tables for each county (consider presenting as Atl and Gulf coasts) with numbers of reported crabs in each landcover classification, and/or numbers of crabs per year. We did not have sufficient time series data of horseshoe crab spawning or ESI/DEM data to provide this information.

-L304-313: This line buries the lead by restating results. Consider recasting to focus on the most important finding, that habitats where HSC were observed most frequently were predicted to be lost under sea level rise scenarios. Then contextualize this finding.-

The introductory paragraph of the discussion was adjusted to highlight potential habitat loss.

L328: Capital S in De Soto Park Fixed
-L337-374: This sentence is at odds with statements in the objectives (L116-118), methods (L1226-129, 170-172), and the discussion (L308-311).Wording changed address this and remain consistent.

-L336-337: The use of habitat preference is not appropriate here, as there was no use vs availability analysis. Further, there was no contextualization of Atlantic coast vs Gulf coast, or ocean vs bay areas, which prevents inference on preference. References to habitat preference were removed and changed to use, to address this concern.
-L336-347: How does the area of hardened and developed shoreLin the two study counties compare to other areas along FL’s coast? We don’t have the data to answer this question, it would require further analyses outside the scope of this study.
-L362-363: Consider removing this sentence.Sentence removed
-L348-360: This section feels out of place, especially considering that temporal variation of results was not presented. Consider removing. This section was removed.
-L361-381: Consider recasting the Discussion, and incorporating this information, as relevant, in Methods and Discussion. While it is critical to transparently acknowledge limitations of any given study, singular dedicated paragraphs can be unintentionally disparaging. Please consider contextualizing results and within that same paragraph, provide the relevant limitations for that finding.

We prefer to be upfront about limitations as all predictive analysis are speculative. We’ve found this format to be viewed positively in other publications with these analyses and strive to be transparent.
-L430-434: The objectives of this study did not include providing a practical approach for identifying areas with high concentrations of species that rely on shoreline habitats for nesting, but rather investigated the potential impact of sea level rise on HSC nesting habitats (L113-114). The methods used did not appear to prioritize elucidating high concentrations of HSC, as the authors note they chose to focus on two counties (L116). Further, the methods are unclear if the analysis accounted for sampling effort, which would be needed to fully understand species abundances and distributions.

We agree this overstated the scope of the study and have adjusted the Conclusion section to be more accurate.

-Data: The data submitted with the manuscript should include metadata that define each of the columns. But concerningly, the data contain sensitive and personally-identifying information. I recommend removing columns U through AI, which contain sensitive information. Apologies, this was uploaded in error. All personally-identifying information has been removed.

6. PLOS authors have the option to publish the peer review history of their article ([what does this mean?](https://track.editorialmanager.com/CL0/https:%2F%2Fjournals.plos.org%2Fplosone%2Fs%2Feditorial-and-peer-review-process%23loc-peer-review-history/1/010f0193c02721c5-454d12e4-b5b9-4e27-ba44-2237fe959925-000000/Kf-E1koPWl_y0YSTZeD4PzyBjjSOCU-KDW1czSgi_XE=189)). If published, this will include your full peer review and any attached files.


If you choose “no”, your identity will remain anonymous but your review may still be made public.


**Do you want your identity to be public for this peer review?** For information about this choice, including consent withdrawal, please see our [Privacy Policy](https://track.editorialmanager.com/CL0/https:%2F%2Fwww.plos.org%2Fprivacy-policy/1/010f0193c02721c5-454d12e4-b5b9-4e27-ba44-2237fe959925-000000/QlCTzu7QKQi6q4fY-r2csOz3n6tRrwKq1dS0UCnE67g=189).

Reviewer #1: No

Reviewer #2: No
