# Supplementary material for: Potential implications of rising sea level on American Horseshoe Crab (Limulus polyphemus) spawning beaches in two Florida counties
Source: PLoS One. 2025 Nov 5;20(11):e0333812. doi: 10.1371/journal.pone.0333812 (PMC12588468; doi:10.1371/journal.pone.0333812)
Supplement: Attachment — Submitted filename: Comments to the Author_05_09_2025.docx [file pone.0333812.s005.docx]

**Comments to the Author**

1. If the authors have adequately addressed your comments raised in a previous round of review and you feel that this manuscript is now acceptable for publication, you may indicate that here to bypass the “Comments to the Author” section, enter your conflict of interest statement in the “Confidential to Editor” section, and submit your "Accept" recommendation.

Reviewer #1: (No Response)

Reviewer #2: All comments have been addressed

2. Is the manuscript technically sound, and do the data support the conclusions?

The manuscript must describe a technically sound piece of scientific research with data that supports the conclusions. Experiments must have been conducted rigorously, with appropriate controls, replication, and sample sizes. The conclusions must be drawn appropriately based on the data presented.

Reviewer #1: No

Reviewer #2: Yes

3. Has the statistical analysis been performed appropriately and rigorously?

Reviewer #1: I Don't Know

Reviewer #2: Yes

4. Have the authors made all data underlying the findings in their manuscript fully available?

The [PLOS Data policy](http://track.editorialmanager.com/CL0/http:%2F%2Fwww.plosone.org%2Fstatic%2Fpolicies.action%23sharing/1/010f019896f82472-477dc538-9dbc-49f8-b0c5-79db4d2b5085-000000/Eej09PSX8UPkED8VbCFsRoHl_tkbdFvFHq5i20YC2Go=223) requires authors to make all data underlying the findings described in their manuscript fully available without restriction, with rare exception (please refer to the Data Availability Statement in the manuscript PDF file). The data should be provided as part of the manuscript or its supporting information, or deposited to a public repository. For example, in addition to summary statistics, the data points behind means, medians and variance measures should be available. If there are restrictions on publicly sharing data—e.g. participant privacy or use of data from a third party—those must be specified.

Reviewer #1: Yes

Reviewer #2: Yes

5. Is the manuscript presented in an intelligible fashion and written in standard English?

PLOS ONE does not copyedit accepted manuscripts, so the language in submitted articles must be clear, correct, and unambiguous. Any typographical or grammatical errors should be corrected at revision, so please note any specific errors here.

Reviewer #1: No

Reviewer #2: Yes

6. Review Comments to the Author

Please use the space provided to explain your answers to the questions above. You may also include additional comments for the author, including concerns about dual publication, research ethics, or publication ethics. (Please upload your review as an attachment if it exceeds 20,000 characters)

Reviewer #1: The revised manuscript by Contrada et al. describes the potential impacts of sea level rise on coastal habitats used by spawning horseshoe crabs. The authors combine sightings of horseshoe crab spawning with sea level rise scenarios and shoreline characterization. Unfortunately, the authors chose not to incorporate a number of edits suggested by the previous reviews, which continue to make the manuscript difficult to interpret. Most importantly, this is related to inconsistent terminologies. A few specific items are mentioned below.

We worked to make the changes that you recommend on the first review, while also accommodating the other reviewers and editors. This may have resulted in some items not being incorporated. We apologize for any continued confusion and hope that we have sufficiently addressed your comments below. We are hopeful that this work is a first step in recognizing the impacts of sea level rise on horseshoe crab habitats in Florida, especially those that are already highly developed. We agree that future work can be done to delineate how exactly certain habitats will progress and change.

• L137-141: If I’m reading this correctly, Figure 1 represents information that was compiled and presented for this article and has not previously been published. As such, it would be more appropriate for Figure 1 to be considered a result, and should be presented in the Results section. In this way, the author can then clarify which datasets were used for which portions of this current study.

We agree, this is a result, we’ve moved Figure 1 to the result section.

• Please include justification for how calculating slope advances the stated goal of the study describing the “potential impact of sea-level rise on horseshoe crab spawning”? I see now the confusion here, and have made changes to clarify. The slope was used as part of the characterization of the spawning habitats in Florida, as there have not been many studies on this, only localized areas. Therefore, we included the description of slope calculation in the Methods section titled ‘habitat characterization’ and incorporated those results into “**Horseshoe Crab Spawning Data and Habitat Characterization**”. The slope was not used explicitly in the sea level rise scenarios, but the Digital Elevation Data (DEM) was used for slope calculations and the sea level rise predictions.

L377: Suggest using ‘anthropogenic’ instead of man-made.

We agree that anthropogenic is a more suitable term but the actual ESI shoreline types are defined as man-made so we think it’s best to keep the definition consistent with NOAA’s standard

<https://response.restoration.noaa.gov/esi-shoreline-types#slideshow-8>

• L380-381: The authors are quoted as saying “Finally, this study did not analyze potential habitat gain or examine potential habitat shifts”. Both reviewers from the previous version of the submitted manuscript pointed out the inconsistency in terminology and requested more consistency. Given the authors own words here, ‘habitat change’ would be an appropriate phrase to use instead of ‘habitat shift’.

We have used change instead of shift in all instances. We have had previous reviewers request shift as a better term which was why we stood by the initial use. But we understand your decision.

• L387-388: The cited study doesn’t seem to support the statement. If I’m reading it correctly, the paper doesn’t address a shift in spawning areas.

It does not describe horseshoe crab changing from one habitat to another, it is unknown how long horseshoe crab had been using marsh habitat so you can’t say they shifted from one to the other but this paper does demonstrate that horseshoe crab will use a variety of once thought to be unfavorable habitat to spawn. For that reason we think it is a valid citation.

• L360-361: I believe this statement overstates the benefits of living shorelines. Please provide a citation for this statement: “One effective solution to protect shorelines from the impact of rising sea levels is to implement “living shorelines”.

Citation: Smith CS, Gittman RK, Peterson CH. Living shorelines enhanced the resilience of saltmarshes to Hurricane Matthew (2016). Ecological Applications 2018;28(4): 871-877.

We believe this citation addresses how living shorelines would reduce the impacts of erosion which is a major concern for sea level rise, but we removed the sentence.

Reviewer #2: PONE-D-24-52275R1
Potential implications of rising sea level on American Horseshoe Crab (Limulus polyphemus) spawning beaches in two Florida counties

In this manuscript, the authors characterized the possible change in spawning habitat at where American horseshoe crab in FL. Using crowd-sourced, community science data, the authors generated a dataset of HSC observations, assigned landcover classifications where HSC were observed, and quantified loss of landcover classifications at three future timesteps. The authors found areas of spawning habitat where HSC were observed in 2017 and 2020 was predicted to be lost due to anticipated sea level rise. The authors propose ways their work can inform restoration and management efforts, and how methodology could be used elsewhere.

I appreciate the authors' care and dedication during revision, which has improved their interesting and valuable work. I have very minor areas of feedback:
-Contextualization of sites: I understand the authors' decision to retain reference to FL counties (vs suggestion to reframe as Atlantic coast/Gulf coast). In Methods (~lines 129-132), please consider adding a brief clause indicating that HSCs in these sites are not expected to differ significantly in behavior (as noted in the response to reviewers document, and cite any supporting information that already exists in the Lit Cited section). Doing so will help reinforce that these are analogous sampling points and not necessarily points chosen to represent different populations/climate change regimes.

We have added text to the Methods - Study Sites, to satisfy this request. “While these sites are geographically separated and the horseshoe crabs are part of genetically distinct populations [25], spawning behaviors were assumed to be similar. The sites were not selected to represent different populations or climate change regimes.

-Line 129: Unnecessary 'n' in sentence.

Fixed
-Lines 118, 133, 225, 232, 348, 356: Unusual punctuation (e.g., space before period, two periods, missing period, missing space, missing period). Please consider a careful review to check for other typos.

We think that this is an artifact for track changes and will be corrected. Thank you!

-Lines 442-444: I believe the complete citation for this should be: Sweet WV, Kopp RE, Weaver CP, Obeysekera J, Horton RM, Thieler ER, Zervas C. 2017. Global and regional sea level rise scenarios for the United States (Technical Report NOS CO-OPS 083). National Oceanic and Atmospheric Administration, Silver Spring, MD. doi: 10.7289/V5/TR-NOS-COOPS-083

Yes! Thank you!

7. PLOS authors have the option to publish the peer review history of their article ([what does this mean?](https://track.editorialmanager.com/CL0/https:%2F%2Fjournals.plos.org%2Fplosone%2Fs%2Feditorial-and-peer-review-process%23loc-peer-review-history/1/010f019896f82472-477dc538-9dbc-49f8-b0c5-79db4d2b5085-000000/v4L6RXV6Yd_bqS5UV_zfGjffEzIt89lch_0RTUsezdc=223)). If published, this will include your full peer review and any attached files.


If you choose “no”, your identity will remain anonymous but your review may still be made public.


**Do you want your identity to be public for this peer review?** For information about this choice, including consent withdrawal, please see our [Privacy Policy](https://track.editorialmanager.com/CL0/https:%2F%2Fwww.plos.org%2Fprivacy-policy/1/010f019896f82472-477dc538-9dbc-49f8-b0c5-79db4d2b5085-000000/RLm_PnhbScwL-AGBCmmCX_p7pU7Picbo9NCnCsmywic=223).

Reviewer #1: No

Reviewer #2: **Yes:** Christy N Wails
